# Costoclavicular-serratus anterior muscle space brachial plexus block provides complete and fast analgesia for patients with upper limb trauma

**DOI:** 10.1186/s13049-021-00887-1

**Published:** 2021-06-02

**Authors:** Qi Chen, Ke Wei, Bin Yang

**Affiliations:** 1 Department of Anesthesiology, Chongqing University Cancer Hospital, Chongqing, China; 2 Department of Anesthesiology, The First Affiliated Hospital of Chongqing Medical University, Chongqing, China; 3 Department of Anesthesiology, The First Affiliated Hospital of Xiamen University, Xiamen, China

Dear editor:

A novel ultrasound-guided regional analgesic technique for limb trauma has been increasingly used in the current emergency medicine. Although various approaches for ultrasound-guided brachial plexus blocks (BPB) have been described in the upper arm anesthesia, identifying which approach would be better for the acute pain management in patients with limb trauma in the emergency room, specifically for fretful children, patients with full stomach, or patients who were drunk, remains controversial. Even though these blocks have been administered as arm anesthesia, none of the present approaches would provide a complete blocking effect with only a single shot of a local anesthetic. The anatomy and branch innervated by the intercostobrachial nerve (ICBN) is significantly different from the medial/posterior upper arm skin innervated by the medial brachial cutaneous nerve (MBCN). ICBN usually originates from T2, with contributions from T1 or T3, whereas MBCN originates from the medial branch and branches out from the cord of the brachial plexus in the infraclavicular fossa [1, 2], but the blocking success rate of subcutaneous ring injection for ICBN is < 20% [3].

With the expansion of point-of-care ultrasound, ultrasound-guided nerve blocking has become readily available and could provide rapid and effective analgesia for acute pain control. The proximal approach of the ICBN block was visualized at the fossa, lying above the anterior chest where the ICBN is delivered from the external intercostal or serratus anterior muscles [4]. In the same plane, brachial plexus bundles are consistently clustered together outside the axillary artery [5]. This costoclavicular space BPB is easily identified using ultrasound and is a more reliable approach for blocking the infraclavicular brachial plexus with BP and ICBN in one ultrasound imaging plane with only a single local anesthetic injection.

**Fig. 1:**
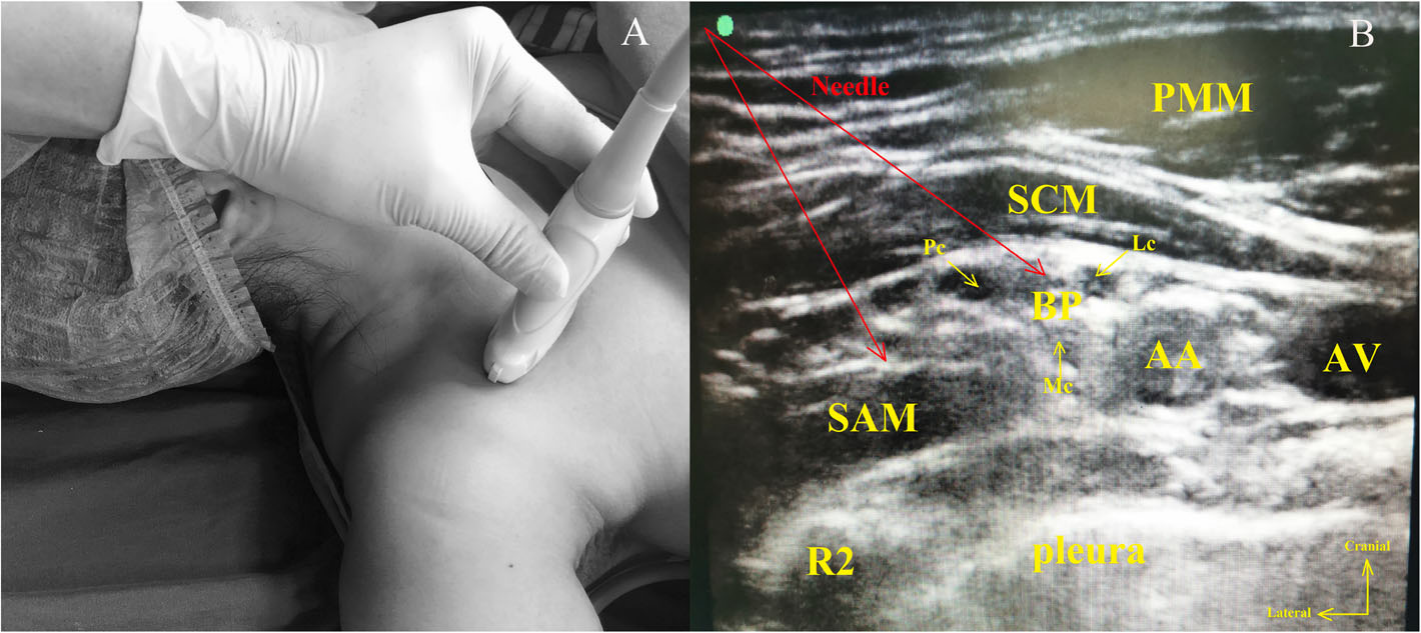
Demonstration of CC-SAS BPB approach. PMM: pectoralis major muscle; SCM: subclavius muscle; SAM: serratus anterior muscle; AA: axillary artery; AV: axillary vein; BP: brachial plexus; Lc: lateral cord; Pc: posterior cord; Mc: medial cord; R2: second rib

Here, we aimed to present our costoclavicular-serratus anterior muscle space (CC-SAS) BPB applications for patients with limb trauma who needed immediate pain control with surgical emergency treatment. This blocking method was applied to three adult patients: left arm burns, left arm long cut, and right upper arm skin laceration. All of them provided written informed consent for all procedures and publications. Patients were positioned supine with the ipsilateral arm abducted (Fig. 1A). A Mindray M7 super ultrasound system with a high-frequency linear array transducer was used for the scan. First, the transducer was placed directly under the mid-point of the clavicle in the transverse orientation and gently moved caudally until the axillary artery and vein were visualized. Then, it was gently tilted cephalad to direct the ultrasound beam toward the CCS, that is, the space between the posterior surface of the clavicle and the second rib. Second, the ultrasound image was optimized until all three cords of the brachial plexus were clearly visualized lateral to the axillary artery and the serratus anterior muscle, clearly showing the second rib (Fig. 1B). The nerve block needle was inserted from the lateral of the brachial plexus to the medial using the in-plane method. Ten milliliters of 0.375% ropivacaine was injected into the superficial serratus anterior muscle and into the center of the three cords of the brachial plexus by retracting the needle to flatten the angle and then advancing the needle. All patients achieved satisfactory pain relief immediately without administering additional local anesthesia or sedatives.

Based on our findings, we concluded that the CC-SAS block could be a beneficial and safe procedure to provide immediate bedside pain control for upper limb trauma and can be easily performed by physicians in clinical practice. However, the feasibility of CC-SAS remains to be confirmed by larger clinical trials and anatomic studies.
